# The Potential of Single-Chain Variable Fragment Antibody: Role in Future Therapeutic and Diagnostic Biologics

**DOI:** 10.1155/2024/1804038

**Published:** 2024-08-09

**Authors:** Getachew Gezehagn Kussia, Tesfaye Sisay Tessema

**Affiliations:** ^1^ Genomics and Bioinformatics Bio and Emerging Technology Institute, Addis Ababa 5954, Ethiopia; ^2^ Institute of Biotechnology Addis Ababa University, Addis Ababa 1176, Ethiopia

## Abstract

The advancement of genetic engineering has revolutionized the field of immunology by allowing the utilization of intrinsic antibody structures. One of the biologics that are being produced by recombinant antibody technology is single-chain fragments variable (scFv). Genes of variable regions, the heavy and light chains that are genetically linked into a single transcript by a short flexible linker peptide, are used to generate this fragment from cellular and synthetic libraries. The specificity and affinity of these molecules are comparable to those of parental antibodies. Fusion with marker proteins and other potent molecules improves their stability, circulation half-life, activity, and efficient purification. Besides, this review comprises construction protocols, therapeutics, and diagnostic applications of scFv, as well as related challenges. Nonetheless, there are still issues with efficacy, stability, safety, intracellular administration, and production costs that need to be addressed.

## 1. Introduction

The mammalian bodies are warm, moist, and contain all the necessary nutrients to sustain their lives. These features also attract most microorganisms that require vital nutrients for their cell division, multiplication, and sheltering. Microorganisms that invade the mammalian body are challenged by multiple defensive mechanisms. For effective protection, the system involves both natural and acquired immunity, leading to a coordinated response. As a result, it clears the body tissues off from non-self-antigens and/or diminishes their damage. An immune system responds to both endogenous and exogenous invaders either via specific or a vast array of mechanisms. In all cases, there might be a sequential surge of cellular as well as biochemical molecules, including, but not limited to, inflammatory mediators, natural antimicrobial proteins, complement components, antibodies, cytokines, chemokines, vasoactivators, vasodilators, cell surface proteins, and hydrolytic enzyme [1, 2, 3].

In the early history of humans, a smallpox virus (supposed to cause an estimated death rate of about 300–500 million victims) has been combated by a technique called variolation. This practice was introduced by the ancient Chinese in the 15th century and involves the use of scabs obtained from recovered individuals to deliberately infect their infants. By doing so, they have successfully saved the lives of their infants, since the clinical cases' mortality rate has dropped from 20% to 1% [2, 3, 4]. Hence, the practice spread to Western Europe and permitted the control of an inevitable cattle plague disease called rinderpest. More importantly, an English physician, Edward Jenner, introduced the new concept of variolation in 1798, which allowed him to discover the clue of cross-protection. Due to the fact, a cowpox scrub efficiently induced a cross-protection against smallpox in humans. This practice later allowed the complete eradication of smallpox from the globe in 1970, and the concept of vaccination was conceived at that time [2, 3, 4, 5]. Following this principle, various scholars, including Louis Pasteur, Daniel Salmon, and Theobald Smith, have developed different vaccines for different infectious diseases [3]. Though all the works of those scholars have proven the efficacy of vaccination, no one understood how it works until von Behring and Kitasato in 1890 demonstrated that a serum derived from animals previously immunized to diphtheria transfers the state of immunity to unimmunized animals [2, 3, 6].

Research in the next few decades proves the fraction of serum components called gamma globulin (now immunoglobulin (Ig)) as a key player in an immune response. Particularly, as Elvin Kabat confirmed in the 1930s, this active molecule in the fraction of immunoglobulin is antibody [6]. Henceforth, antibodies attract the attention of various scholars as they can capably exhibit activities such as neutralization, precipitation, and agglutination toward their specific antigens. Unlike innate immune cells, antibody responses produce an immunological memory in animals encountered with a specific pathogen, which is the concept that forms the basis of vaccination. Moreover, they can be modified to take on various formats and restructured for a range of applications against a diverse array of antigens or target molecules. Such property makes them be leveraged for numerous purposes, such as research and the development of diagnostics and therapeutics [7, 8].

Antibodies can be polyclonal, monoclonal, or recombinant based on the nature of their production. Though each has its own intrinsic limitations, they may excel at the advantages they possess in their specific area of application. Historically, the first polyclonal antibody directed against diphtheria toxin (DT) was produced in 1890 by von Behring and Kitasato [6, 8]. Though these antibodies are cheap to produce, they have limited applications in therapeutics and diagnostics because of the risk of immunogenicity and their low specificity. Hybridoma technology, established by Köhler and Milstein [9], allowed the production of monoclonal antibodies (mAbs) from fused immortal myeloma cells with B cells derived from mouse spleen [8, 10, 11]. However, this technology has been compromised with some limitations, like the instability of the aneuploid cell lines, the inability to produce human antibodies, the low efficiency of B lymphocyte fusion with myeloma cells, and the experimental animals subjected to immunization [12, 13].

Recently, progressive advancements have been made based on the understanding of immunoglobulin organization, its structure, and the diversity generated through somatic recombination and hypermutagenesis of variant genes [14]. Knowledge of these enables scientists to produce IgG-like natural antibodies in mammalian bodies. However, this method is restricted by some distinct problems, including high production costs and long processing times [15, 16]. Meanwhile, mAbs are epitope specific, so they can be recombinantly tuned to target specific molecules involved in underlying pathological conditions. This property sets mAbs apart from polyclonal antibodies in addressing therapeutic and diagnostic limitations related to them [8]. Moreover, mAbs can be generated via chimeric or humanized hybridomas and can recognize human epitopes, so they overcome the risk of immunogenicity. Thus, humanized antibodies have been successfully used in treating a wide range of human ailments, including cancer, autoimmune disorders, cardiovascular diseases, and several neurological disorders [17, 18]. However, mAbs are still inadequate to mark all targets because of the very cryptic nature of some antigenic molecules involved in particular pathological milieus. In addition, the penetration of mAbs into solid tumors and their intratumoral distribution are limited. This influences the potency of their application in molecular imaging and the therapeutic arena [19]. Thus, researchers initiated a search for alternative antibody layouts [7]. The recombinant antibody technology enables them to produce antibodies with different modifications that have high affinity and specificity for other biological molecules.

Recombinant antibody fragments are a current top area of research that excels with different properties over full-length mAbs. The fragment molecules can be produced more economically and straightforwardly through techniques of antibody engineering, molecular cloning, and even enzymatic methods compared to conventional antibodies. This new paradigm creates a platform for a new therapeutic discipline with the capacity to target molecules involved in specific pathological disorders, different from the previous technology schemes. In this regard, a variety of antibody fragments have been generated in unlimited quantities for various purposes in research, therapeutics, and diagnostics [18, 20]. As documented by a lot of literature, several antibody fragments currently exist. The most well-known ones are single-chain fragment variable (scFv), single-domain antibodies, antigen-binding fragment (Fab), and the crystallizable fragment (Fc) domain [21]. Besides its smaller size, scFv Ab comprises both variable heavy (VH) and variable light (VL) chains, which can be obtained from various libraries and determine the complementary determining region (CDR) of the antibody. It can be engineered into larger multimeric forms like bispecific, trivalent bispecific, tetravalent bispecific antibody derivatives, and conjugated forms for numerous applications. Thus, this review paper aims to discuss some recent progress and applications of scFv in research for future therapeutic and diagnostic purposes.

## 2. Single Chain Fragment Variable Antibody (scFv)

### 2.1. Design Variations on Single Chain Fragment Variable Antibody (scFv)

**Figure 1:**
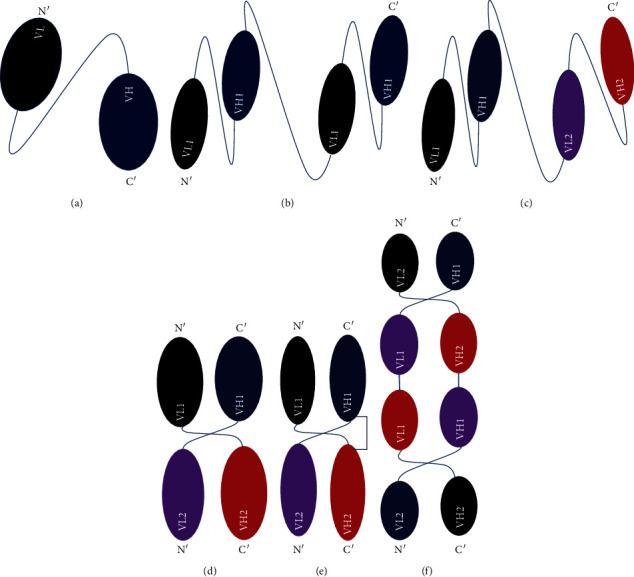
A graphic summary of the scFv antibody and its related platforms, partially adapted from Bates and Power [21]. (a) The scFv antibody format: The C-terminus of VH is joined to the N-terminus of VL via a flexible peptide linker that is rich in glycine and serine residues. (b) A bivalent monospecific tandem of scFv, made of two identical scFvs, is joined via a helical linker. (c) A bivalent bispecific scFv made of two different scFvs is joined via a helical linker. (d) A bispecific diabody scFv format constitutes two separate chains, each with VL and VH from different antibodies, in a head-to-tail arrangement. (e) A bispecific dual DART® format created from two distinct polypeptide chains tethered together via the interaction of a noncovalent and a disulfide bond. (f) A tetravalent bispecific molecule called TandAb is made of two diabodies linked in a linear arrangement.

A scFv antibody is made from genes of variable regions, the heavy and light chains, which are genetically tethered in a single transcript via a short flexible linker peptide (Figure 1(a)). It is usually oriented in a way that allows it to bind the target antigen with the same specificity and affinity as the parental antibody. Its average molecular weight is 27 kDa. Hence, it is acknowledged as the smallest functional immunoglobulin unit of all antibody fragments with VH and VL domains. Both VH-linker-VL and VL-linker-VH arrangements can produce useful scFvs, though in some cases, scFvs perform better in one conformation than another [22]. The length and sequence of the linker peptide, however, play a significant role in determining the binding feature, specificity, folding, and solubility of scFv antibodies [23, 24]. The linkers are mostly within 15–20 amino acid sequences and rich in glycine and serine residues, nevertheless, it can be optimized up to 35 amino acids [25]. The linker spans a distance of 3.5 nm (35 Å) between the carboxy- and amino-termini of the two variable domains [26]. The glycine–serine-rich linker sequences are the most commonly used due to their flexible nature. *In vitro* studies, however, show that those repeated sequences carry problems in PCR-based experiments, appear immunogenic, and display less stability than nonrepetitive linkers [23]. Moreover, serine, particularly and other charged residues such as glutamic acid and lysine (as EAAAK)2, if included in linker sequences, have improved the rigidity and solubility of a scFv [27, 28].

Typically, the peptide linker is designed with a chosen orientation. A linker that is too long promotes the proteolysis of the two domains, whereas a linker that is too small prevents the physical annealing of the two domains and is used to create multimers such as diabodies, tribodies, and bispecific antibodies [29]. The linker sequence design, called 15-mer (G4S)_3_, was initially employed by Huston et al. [30] and has since been used in a number of studies. It is a multimer of the pentapeptide GGGGS [31, 32]. Thus, the (G_4_S)_*n*_ linker amino acid sequence motif is used as a parental scaffold, where the 18-mer GGSSRSSSSGGGGSGGGG and the 20-mer (G_4_S)_4_ are some commonly used multimers [33, 34, 35]. As documented well in many articles, different formats of scFv-based antibody structures have been generated based on the number of scFv antibodies and peptide linkers adopted. For instance, a tandem scFv joins two or more scFvs through helical peptide linkers in the NH2-VL1-VH1- (linker-VL2-VH2) *n*-COOH configuration in a single transcript. These produce a biologic with a bivalent and/or bispecific binding site that targets a particular antigen with greater avidity or targets two discrete antigens simultaneously (Figures 1(b) and 1(c)). Moreover, it may even target albumin, which increases the circulation half-life of the fragment molecule [36].

scFv antibodies can be modified further by multimerization technology that increases their molecular weight, affinity, and sensitivity [37, 38]. It can also be modified by the interaction of two different chains from two different antibody molecules to form bivalent dimers called diabodies (Figure 1(d)) [36]. In one of these formats, called dual affinity re-targeting proteins (DARTs), two different chains are used to generate bispecific bivalent dimers, where the first chain contains VH from Antibody 1 and VL from Antibody 2, and the second chain contains VH from Antibody 2 and VL from Antibody 1 (Figure 1(e)). Then an interchain disulfide bond is added between the two polypeptides that improves the stability of the molecule and reduces the aggregation degree of homodimers [39]. Moreover, by joining three or more variable domains in a single chain, it is possible to produce trivalent, tetravalent (Figure 1(f)), or pentavalent structures in a manner similar to that of a diabody [40].

### 2.2. Advantages of scFv Antibody

A scFv antibody has several advantages over full-length mAbs with its intrinsic features, like size, immunogenicity effects, expression, and production system. The smaller size of scFv antibodies (Mwt of 27 kDa), i.e., one-fifth less than that of complete antibodies (150 kDa), allows for the easy tissue penetration of tumors and access to cryptic epitopes. Furthermore, its rapid clearance from blood and nontarget tissues is enhanced due to its small size. More importantly, easy production and manipulation in a microbial expression system, which yields higher doses within a short period of time with lower costs, are possible with the scFv antibody molecule. The scFv are less immunogenic, as they lack an Fc domain responsible for bystander activation of antibody effector functions. Moreover, it possesses the same antigen-binding capacity as that of the parental antibody and can even be used in conjugation with other potential molecules that improve its activity, stability, and affinity to bind target molecules with greater propensity [13].

### 2.3. Generation of scFv Antibody

**Figure 2:**
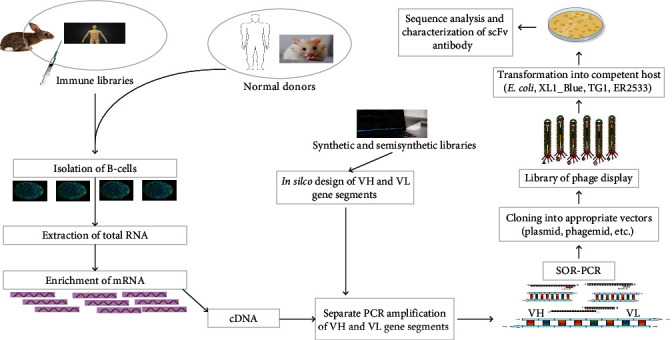
A summary of the basic procedures for creating the scFv antibody. A diverse repertoire obtained from the reshuffled variable gene segments can be derived either from naïve B cells, activated B cells, and/or synthetic repertoires as rearranged variable gene segments *in silico*. After mRNA enrichment, the scFv repertoires are assembled via SOR PCR and then cloned into a phagemid vector in order to be expressed on the surface of the phage as a scFv library. The phage library incubated with the target ligands is then subjected to washing steps to remove all unbound phages. All bound phages are eluted and propagated in *E. coli* and could be used for further rounds of selection to get the specific binders.

The protocols for the generation of scFv antibodies are essentially based on the previous procedures developed by two separate laboratories [30, 41]. However, slight modifications have been used in diverse studies, with trivial differences in the initial cell preparation and construction of the cloning library. The protocols are presented as follows (Figure 2).

#### 2.3.1. Isolation of mRNA and PCR Amplification of VH and VL Genes

Using the genes extracted from murine hybridoma cell lines, Huston et al. [30] created the first scFv antibody. Currently, several cellular and synthetic antibody libraries comprised of immune and universal libraries have been used as raw materials for the generation of diverse antibody formats (Figure 2). Immune libraries are generated from the samples from immunized animals or humans' tissues or blood such as bone marrow, peripheral blood mononuclear cells (PBMCs), spleens, hybridoma cell lines, and any cellular components [31, 42, 43]. On the contrary, universal libraries are labeled for general use and classified as naïve, synthetic, and semisynthetic libraries. Naïve libraries are derived from reshuffled Ig V genes of nonimmunized donors and are combinatorial in nature, where the heavy and light chains are randomly combined in the course of library construction. Synthetic libraries are developed using the art of molecular biology techniques with the aim of potentiating recombinant antibodies [44]. Semisynthetic libraries are classically derived from combined natural and synthetic antibody sequences [45]. With both immunized and nonimmunized donors, the gene segments encoding the VH and VL antibody domains are produced via the isolation of total RNA and its enrichment. The next step is reverse transcription to produce cDNA, which is used as a template for the PCR amplification of the related genes (Figure 2). Using forward and reverse primers, amplification of cDNA fragments creates a huge library with a wide variety of VH and VL antibody genes. Several investigators follow the standard PCR steps, but they slightly alter them depending on the study's goal and the PCR optimization parameters [31, 42].

#### 2.3.2. Introduction of Linker Peptide and Cloning of Full Length scFv Domains

The segment of DNA linker encoding a pentadecapeptide (Gly4Ser)_3_ amid VH and VL PCR amplicons will be done with a pair of primers designed for those. The linkers are separately added behind and in front of each variable domain first. Then a two-step overlapping PCR called splicing by overlap extension (SOE-PCR) is used to assemble them. VH and VL domains should be present in equal amounts in the assembly PCR, but the linker primer might be lowered to prevent the preferential amplification of one domain. The PCR products would then be verified through gel electrophoresis, and the appropriate fragments would finally be subjected to sequencing platforms. To add a restriction site to the product of the PCR-assembled domains, outer primers are used at the time of PCR assembly, which may be either primers used to amplify the two domains or a new pair of extension primers. By doing so, both ends of the scFv sequence might be extended, either to independently amplify the assembly of the domain sequence or to add a restriction site, which is significant during library creation. As a consequence, a full-length scFv of either orientation VH-(Gly4Ser)_3_-VL or VL-(Gly4Ser)_3_-VH would be generated and cloned with the help of the specific designed primers [42].

#### 2.3.3. Cloning in Shuttle Vectors, Phage Display, and Transformation

The purified full-length scFv antibody fragment and vector used for cloning (e.g., pAk100, PJB/2, pCOMB3X phagemid, etc.) are subjected to endonuclease restriction enzymes. In some cases, the cut vectors are laid open for the dephosphorylation step (e.g., with calf intestinal alkaline phosphatase) in order to eliminate any risk of their religation [34]. The phagemids with engineered small fragment molecules (scFv fragments) are then ligated into phage display vectors in the presence of ligase enzymes [42]. The phagemids with engineered small fragment molecules (scFv fragments) are then ligated into phage display vectors in the presence of ligase enzymes [40]. The phage display technology was developed by Smith [46] in 1985 in order to encode different recombinant peptides into the phage coat protein as fusions to be displayed on the surface of the virion [47]. The gene encoding the scFv antibody of interest is incorporated inside the bacteriophage and attached to the C-terminal region of DNA, while the displayed peptide would be attached to the exterior N-terminus of the gene. Several phage particles are used for phage display, but the most popular one is the filamentous phage M13, while other phages, such as Lambda, T4, and T7, are also used. M13 is nonlytic to host bacteria and secretes the produced phage particles through the bacterial cell envelope. M13 owns two structural proteins used for the expression platforms of antibody fragments of interest: genes III and VIII. Due to the lower number of displayed peptide copies on the surface of the phage, the gene III protein is widely used [48].

**Figure 3:**
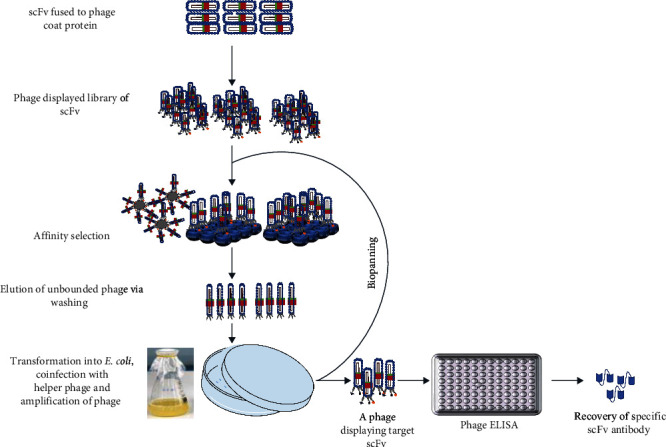
Schematic sketch of *in vitro* selection protocols by phage display biopanning. Gene segments encoding the desired scFv are fused with phage coat protein-coding genes. The library of phages displaying scFv antibody is subjected to affinity selection via incubation with antigen existing in its native form, either immobilized on magnetic beads or solid surfaces. All unbound phages (weakly bound) are removed by washing, while the firmly bound phages are recovered by changing pH conditions. The coinfection of *E. coli* cells with helper phages releases a new phage particle used in the ensuing rounds of panning cycles, which comprise 3–4 rounds. Finally, phage enzyme-linked immunosorbent assay (ELISA) is used for enrichment of binders, with subsequent recovery of the desired scFv antibody.

By the standard protocols of relevant PCR, a scFv antibody-encoding segment is fused to the gene for the phage coat protein. The linear PCR product is then amplified and circularized to give a viral genome that is transformed into *E. coli* cells such as XL1-Blue, TG1, and ER2537 either by electroporation or chemically induced competency (Figure 2). To release the cloning vector harboring the required segment, the competent bacteria are infected with helper phages such as VCSM13, M13KO7, etc. *E. coli* cells then produce a great number of phages exhibiting a library of diverse peptide sequences, mostly 10^6^–10^11^ in size, based on the library type under investigation. From these, libraries of antibody variable domains are constructed and screened to find peptides that have high affinity for specific molecules like enzymes, antibodies, and surface receptors of the given cell. These *in vitro* selection processes for peptides of interest are called biopanning [47, 48]. Through this process, the avidity, affinity, and binding kinetics of small fragment antibodies to the antigen of desire are determined. The stepwise procedure includes incubation of the display library with ligands that are bound to a solid support (beads, membranes, etc.); release of unbound phage via washing buffers; elution of bound phage; and reinfecting it with an *E. coli* cell (Figure 3). At the end, a library of phages, each phenotypically expressing a single antibody fragment on its surface, is generated. Numerous cycles of binding (four to six in most cases) and amplification will be used to select antigen-specific clones of phages displaying antibody fragments that compactly bind to the target molecule. Finally, each clone is marked by DNA sequencing, which reveals their different CDRs [47]. For instance, phage biopanning was used to select specific scFv clones constructed from a human synthetic scFv antibody library against human membrane-bound fragment of cell adhesion molecule 1 (MF-hCADM1) and mouse membrane-bound fragment of cell adhesion molecule 1 (MF-mCADM)-BSA (bovine serum albumin) that are immobilized on magnetic beads [49].

#### 2.3.4. Expression of scFv Antibody

Display technologies facilitate the rapid screening of multiple antibody fragments in a single experiment and produce pure antibody molecules with high specificity [48]. Small fragment molecules have been successfully displayed using a variety of expression systems including the eukaryotic cell expression system (yeast and mammalian cell-based), the prokaryotic cell expression system (bacterial cell-based), and the cell-free expression system (ribosome displays) [21].

*(1) Prokaryotic Cell Expression System*. Because prokaryotic cell expression systems are inexpensive and can grow quickly in lab settings. Hence, they produce high biomass and protein products during large-scale cultivation in bioreactors. As a result, they are preferred for the expression of smaller antibody fragments [50]. *E. coli* as a model bacterium has been widely used due to its simple genome and ease of analysis. It possesses only a single copy of most genes. Different strains of *E. coli* are used under different conditions to achieve the highest protein expression. For instance, with *E. coli* Origami (DE3), the highest fusion protein expression was achieved in 4 hr after induction by 0.1 mM isopropyl *β*-D-1-thiogalactopyranoside (IPTG) at 37°C [51]. However, as the bacterium is Gram-negative, it possesses two membranes external to the inner membrane: the cytoplasmic membrane and the outer membrane. An outer membrane is important for an extra layer of protection. However, it is problematic to assemble engineered proteins from genes cloned into the bacteria since it deters protein secretion. Expression of proteins in *E. coli* cytoplasm produces high yields, but it is challenging due to inconvenient reducing conditions. This condition impairs two intrachain disulfide bonds that determine the antigen-binding activity and proper folding of scFv antibodies. As a result, an expressed product attains the form of inclusion bodies. Hence, additional solubilization protocols by various denaturing agents and further *in vitro* refolding steps after purification are required. These are tedious, inefficient, time-consuming, and costly [52, 53].

To moderate these challenges, the gene that facilitates the co-expression of chaperones with the protein of interest is inserted along the scaffold gene segment. By doing so, the protein will gain its correct folding in the reducing environment. Others fuse the target protein to a short signal peptide (i.e., the N-terminal leader peptide). This peptide directs the protein of interest to an oxidizing space called the periplasm. Periplasmic space is presumed to contain essential proteins such as chaperones and disulfide isomerase. These are important for the formation of disulfide bonds that give proper folding to recombinant antibodies. However, the method yields low protein, so if effective folding of antibody fragments is possible, cytoplasmic expression is favored. Yet there is an upsurge of interest by some investigators in gram-positive bacteria (e.g., Bacillus) that lack an outer membrane [54, 55].

*(2) Eukaryotic Cell Expression System*. For the eukaryotic cell expression system, the most commonly utilized strains in molecular biology are single-celled yeasts from kingdom fungi. These include *Pichia pastoris* [53] and brewer's yeast, *Saccharomyces cerevisiae* [56]. They grow faster in culture and offer higher levels of expression than any other alternative expression platforms, such as baculoviruses and mammalian tissue culture. They also share many advantages of eukaryotic cells, including protein processing, protein folding, and posttranslational modifications. In both, *S. cerevisiae* and *P. pastoris*, the complementation, gene replacement, and gene disruption transformation methods are used equally; nevertheless, the level of protein expression is 10- to 100-fold higher in *P. pastoris* than in *S. cerevisiae*. The vectors used for the production of heterologous proteins in both strains are episomal plasmids (YEp), integration plasmids (YIp), and centromeric plasmids (YCp). Expression of scFv antibodies can be either intracellularly or extracellularly, depending on the secretory pathway, if signal sequences are genetically fused to the proteins of interest [57]. For instance, the secretion signal sequence MAT*α* factor prepropeptide (MF-*α*1) for *S. cerevisiae* has been used successfully. A scFv fused with fluorescent proteins has also been used efficiently to label proteins in *S. cerevisiae*, which will be a significant tool for functional and localization analysis of the yeast proteome in the future [58].

The *P. pastoris* strains used for the expression of scFv antibodies have defects either in the HIS4 gene, which codes for histidinol dehydrogenase, or the ARG4 gene, which codes for arginosuccinate lyase, or in both genes. All the strains can grow on a complex general medium called yeast peptone dextrose. But minimal media are used for the selection of recombinant strains with His+ or Arg+ prototrophs among his4/arg4 mutant strains transformed with a plasmid carrying the wild-type gene [59, 60]. The general features common to all plasmids used for scFv antibody expression in *P. pastoris* include the inducible promotor 5′PAOX1 from the AOX1 gene, the multiple cloning site (MCS), terminators (poly A sequences), selectable markers, the 3′AOX1 sequence, the origin of replication, and antibiotic resistance genes [61, 62]. In addition, unique restriction endonuclease sites allow for the production of linearized plasmids via integration at the AOX1 locus (NotI and BglII) and/or at 5′PAOX1 (SacI and BstXI) and HIS4 (SalI and StuI) by gene replacement may also be included [63]. However, additional features can be included in some expression vectors on the basis of the study scheme, which might serve as tools for specialized functions.

Although yeast surface display is a more popular approach to expressing antibody fragments like scFvs or Fabs, other recently evolving display systems, such as insect and plant cell-based expression systems, have been utilized. Yet their posttranslational modification process produces a structure called glycan that is different from that of a human. This poses the risk of eliciting an immune response in humans and limits their therapeutic uses. Thus, mammalian cell-based systems are acknowledged for use to avoid the risk of immunogenicity since they can perform glycosylation patterns, proper folding, and posttranslational modifications analogous to those in humans. Moreover, they can be used for ease of large-scale production to harvest high volumes due to their tendency for high-level expression of therapeutic proteins. Yet their procedures are tiresome, time-consuming, and costly [64].

**Figure 4:**
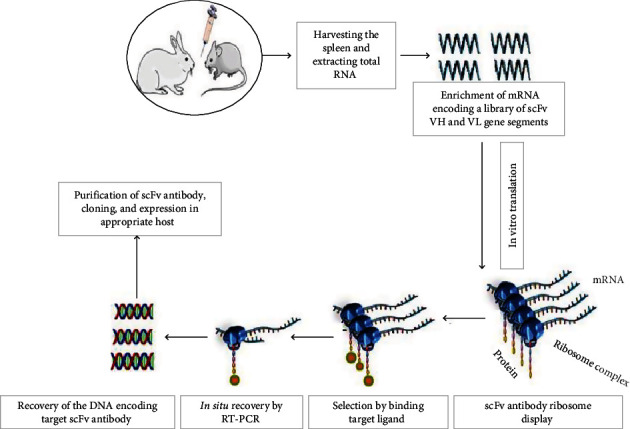
Schematic picture of *in vitro* ribosome display. The DNA library for scFv antibodies of interest is generated from immunized donors. After mRNA enrichment for the library of VH and VL segments, an *in vitro* translation with a ternary protein–ribosome–mRNA complex generates a biologic of desire, which is selected by a target ligand. Further, *in situ* protocols are followed to recover the DNA library of scFv under study and amplify it for a further selection cycle or postselection analysis. Hence, a DNA library can be amplified once or more via *in-vitro* coupled transcription and translation to form mRNA, the associated biologics, and the ribosome complex (PRM complex) that is selected by binding to antigen immobilized on the solid supports.

*(3) Cell-Free Expression System: The Ribosome Display Technology*. This is a scheme of *in vitro* selection protocols for potent antibodies against the desired ligand, based on the inherent physical association between phenotype and genotype [65, 66]. It is primarily designed to mitigate the drawbacks of cell-based and phage-based display methods by producing ternary protein–ribosome–mRNA complexes *in vitro*. In the selection process, the mRNA hybrid protein will bind to an immobilized ligand, exposing the mRNA in the complex for reverse transcription *in vitro* and producing a cDNA sequence that is finally amplified via PCR. Further expression procedures are conducted via panning cycles of 3–5 to obtain a specific antibody fragment (Figure 4). A library of DNA encoding a specific library of the desired product is fused to a spacer sequence voided by a stop codon. During *in vitro* translation, the spacer sequence is linked to the peptidyl tRNA and occupies the ribosomal shaft. By this, the properly folded desired protein is protruding out from ribosomal complexes to bind to an immobilized ligand on a solid surface. Nitrocellulose, column matrices, magnetic beads, polystyrene tubes, and 96-well microtiter plates are some commonly used surface materials. During the washing step, all nonbound complexes will be swept away. But the mRNA of the bound complexes that present the required polypeptide via *in situ* RT-PCR will be recovered. Finally, all identified antibody clones are confirmed via ELISA, and, if necessary, further *in vitro* affinity maturation steps will be carried out to increase the stability and/or affinity of the small fragment molecules [67, 68]. Individual binders are then sequenced and further characterized biochemically. These methods efficiently screen out large libraries of coding DNA and select high-affinity binding sites because no cell culture is involved, compared to other display technologies that are limited by transformation efficiency and posttranslational modifications. A scFvs constructed from the spleens of immunized mice to neutralize Zika virus envelope (E) protein was efficiently generated by the ribosome-displayed system [69]. However, a prominent drawback in this ribosome display protocol is the difficulty of active ribosome levels accessibility used for the generation of the library, which depends on the library size [66].

#### 2.3.5. Purification of scFv Antibodies

During their production cycle and expression systems, antibody fragments can be contaminated with impurities. These can be endotoxins, endogenous viruses, host cell proteins, DNA, and other aggregates that should be eliminated by an efficient purification approach. Preliminary steps by treatment with ammonium sulfate, polyethylene glycol, ethacridine, and octanoic acid can be used to increase the concentration of the antibody fragment [18]. However, the purity, selectivity, reproducibility, and yield of precipitation can be influenced by several factors, like temperature, time, pH, and the rate of salting. Bacterial strains used for expression also influence the formation of disulfide bonds in the cytoplasm. For instance, the SHuffle® strain of genetically engineered *E. coli* can form disulfide bonds properly in its cytoplasm since it is trxB-(thioredoxin reductase), gor-(glutathione reductase), and overexpresses the cytoplasmic DsbC chaperone. Thus, shuffle® produces a higher soluble anti-HER2 scFv with enhanced solubility and biological activity than the BL21 strain of *E. coli* [52].

Differences in the expression system also subject the fragment molecules to various purification steps. Hence, antibody fragments expressed in *E. coli* cytoplasm are first freed by lysing the cells, solubilized with different detergents, and refolded to their proper conformation, unlike those secreted through the bacterial envelope from the periplasmic space. For instance, affinity chromatography under native, denaturing, and hybrid conditions efficiently purifies the protein IP-10 (anti-HER2 scFv). But when hybrid conditions are combined with the urea/imidazole method, the highest level of purity was achieved for the fusion protein [53]. Moreover, antibodies can be separated based on their general, predictable structure, size, and chemistry, as well as by interactions of proteins that have a stronger affinity for specific classes of antibodies, such as IgG, IgM, IgA, IgD, and IgE, and the fragment structures derived from them. However, the most common purification approach used in small fragment molecules is antigen-specific capture, where an antigen of interest that selectively binds a particular antibody, scFv, is immobilized to a solid support such as a resin column or beads. In certain circumstances, it is difficult to immobilize the capture antigen onto a solid support. In these cases, tagging proteins are genetically fused with antibody molecules. Thus, when the gene is cloned and inserted into an appropriate vector, a hybrid gene is expressed, and the antibody synthesized is tethered to the tag molecule, which is later purified along with it [55].

In the protein-specific purification approach, various analytical tools have been used, and the most common is affinity chromatography, which utilizes different protein molecules derived from various microorganisms. For example, protein L (from *Peptostreptococcus magnus*) was used to capture a vast range of fragment antibodies as it specifically targeted a variable region of k1, 3, or 4 light chains. But it is unable to capture fragments derived from a *λ* mAb and the *κ* 2 subfamily. As a result, *k*- and *λ*-selective resins are currently constructed and commercially available as alternatives [70, 71]. Further, bacteria-originated proteins, such as *staphylococcal* Protein-A (SpA), *streptococcal* Protein-G, and *Mycoplasma* Protein-M, have also been used [72, 73, 74]. In full-length mAbs, the Fc domain area binds Protein A with high affinity and specificity. However, small antibody fragments do not possess this domain. Hence, the absence of the Fc domain in small antibody fragments obscures effective purification by those proteins (i.e., Protein A or Protein G). In these cases, selective capture by specific antigen ligands fixed on a solid surface or ligands against affinity tags is recommended as a substitute approach. As mentioned earlier, affinity tags are purposely fused to the fragment molecules by a cleavable linker. The most widely used types of tags are short peptide tags, including His tag, FLAG tag, and Strep tag. However, the longer tags cover a full-length protein such as glutathione-S-transferase (GST), maltose-binding protein (MBP), and protein A, derived from Schistosoma *japonicum*, Staphylococcus, and *E. coli*, respectively, are also used [55, 75].

In all circumstances, affinity tags are captured with their complements immobilized on the metal surfaces and thereby allow fragment antibodies to be selectively purified through affinity chromatography, such as immobilized metal affinity chromatography, regardless of their Ig germline family. For instance, His-tag tightly binds to nickel ions, so His-tagged antibody fragments are purified on a column to which Ni2+ ions are immobilized by a metal chelator. Then, antibody fragments are eluted by changing the buffer conditions, which reduce the interaction between nickel ions and the His tag [52, 55]. Limitations to these protocols come from proteolytic cleavages that aim to remove affinity tags. Hence, incomplete cleavages in some cases may leave residual amino acids that lead to fragment aggregation, misfolding, and immunogenicity problems. As a result, multimodal purification approaches through various chromatography platforms in combination with each other, conjugation to affinity tags, and nonchromatographic methods have been recommended to attain the high-purity antibody fragments for desired applications [21].

## 3. Applications of scFv Antibody

After their discovery, antibodies have attracted the interest of various immunologists, like Paul Ehrlich, who denoted antibodies as “magic bullets” against non-self-entities. In his model, Paul considered antibodies as molecules that comprise several attachment sites for foreign material and for the activation of the complement pathway. At present, antibody-based immunotherapies and diagnostics are greatly esteemed in oncology, autoimmune disorders, neuropathy, chronic inflammatory diseases, and various infectious diseases [18].

### 3.1. Therapeutics

According to the dataset of antibodies in the watch article series, virtually 1,200 therapeutic antibodies are in clinical trials at present. Of these about 175 are under regulatory review and around 140 are undergoing evaluation in pivotal Phase 2, Phase 2/3, or Phase 3 clinical trials called “late-stage” [76]. Moreover, more than 700 mAbs are projected for preclinical and clinical development. As per the United States Food and Drug Administration (US FDA), 106 of these mAbs are approved for cancer therapy [76]. Yet conventional mAbs are limited in their capacity to recruit T cells due to their lack of an FcR. Thus, most scFv-based layouts are amended into multispecific designs to attain the potential to tether an intracellular signaling domain (CD3+) of T cells to a specific tumor antigen family. The well-known identified potential tumor-specific antigen family includes epidermal growth factor receptor (EGFR), vascular endothelial growth factor (VEGF), and hepatocyte growth factor (HGF). By so doing, T cells are activated via the T cell receptor (TCR) complex, independent of their major histocompatibility complex (MHC) role, to perform their effector functions [77]. The most bispecific scFv formats are BiTE (bispecific T cell engager) and CAR (chimeric antigen receptor) T cell therapy, and details of multispecific antibody formats are illustrated well in Kaplon et al. [76] and Mullard [78] review articles. All bispecific formats possess an affinity and specificity higher than monovalent fragments. Thus, due to their miniature size, scFv antibodies are mostly used in conjugation with other molecules to generate a protein with high affinity and specificity for the targeted treatment and uncovering of several pathological diseases, like cancer, autoimmune syndromes, neurodegenerative diseases, etc. [18, 49, 79]. Most scFv-based therapeutic formats are designed for application in cancer and autoimmune diseases, and there are abundant investigations under various phases of clinical development that target well-established antigens on the surface of tumors, as discussed well in a review by Houen [18] and Lou and Cao [77].

Starting with OrthocloneTM (OKT3), a mAb therapy developed for treating organ transplant rejection in 1986, the US FDA has licensed more than 106 mAbs presently. In addition, greater than 60 fragment antibodies alone and 150 antibody fragments based on multispecific formats, particularly BiTE and CAR T cell therapy are under investigation in various clinical trials [77]. Most of them are for both solid tumor cell antigens, autoimmune diseases, and hematological cancers. Of fragment-based formats, most bispecific antibodies and CAR-T cells have been approved for cancer therapy in years between 2021 and 2022, as described in Table 1. For instance, an anti-CD3 scFv called tebentafusp (Kimmtrak®) is the only bispecific gp100 peptide-HLA-directed CD3 T cell engager antibody approved by the US FDA for the therapy of metastatic uveal melanoma in adult patients [76]. Furthermore, about six fragment-based CAR-T cells have also been licensed for the therapy of cancer (Table 1), and the development of antibody fragments for infectious disease therapy is still in the infant stage, probably due to their lack of effector function [21]. Yet no scFv antibody has been approved for use in infectious diseases, but there are growing investigations under various clinical trials in these areas.

#### 3.1.1. Cancer Immunotherapy

Today, several studies with scFvs as fusion proteins have been generated against tumor cell surface antigens as well as molecules associated with cancerous cell proliferation, migration, and apoptosis. CAR T cell therapy, which syndicates the cytotoxic potential of a T cell to the fixed targeting of an antibody in a single gene transcript, has been approved as a powerful tool. The underlying structure of the construct can be modified to include a costimulatory domain that improves T cell survival and its target-directed potential. The most targeted molecule is CD19, which is principally expressed on the surface of leukemia, lymphoma, and most solid cancers [93, 94]. Hence, this technology has previously been used to develop therapy for B-cell malignancies. Though their clinical trials have not yet proven satisfactory, extensive research is currently focused on fighting the challenges limiting the usage of CAR-T cells and their optimization. These schemes involve the use of specific antibody fragments like scFv, which allow easy tissue penetration of therapeutic constructs and potentiate the efficacy of the construct molecule against leukemia and other malignancies. Hombach et al. [93] constructed a CD19 CAR T cell that is readdressed against ErbB2+ carcinoma cells by fusing a protein, herceptin-derived anti-ErbB2 scFv 4D5, linked to the CD19 exodomain. This CD19-4D5-fused scFv molecule potentiates CD19 CAR T cells to target ErbB2+ cancer cells and repress tumor development by displaying a 100-fold greater selectivity toward cancerous cells than healthy fibroblasts, which was not seen in ErbB2 CAR T cells alone (without scFv fusion). These mean CD19 CAR T cells are guided by a CD19-scFv engager protein to precisely target and attack the figured cancer cells.

Another scFv-based cancer therapy is a BiTE, which is designed to retarget T-cells toward cancer and cancer-associated cells in the tumor microenvironment. The format incorporates two scFv fragments from two different mAbs allied via a short flexible linker. This allows the construct to freely rotate and fold to amenably interact with target receptors on the two opposing cell membranes: the cytotoxic T-cell and tumor cell, with consequent stimulation of T-cell initiation. A blinatumomab composed of an anti-CD 19 and anti-CD 3 scFv in VL-linker-VH orientation was developed for the treatment of B-cell precursor acute lymphoblastic leukemia and was previously approved by the FDA in December 2014 [95]. Underlying this format, a number of preclinical investigations have been conducted today to develop a potential therapy for solid tumors. In the study by Liu et al. [96], an engineered protein, protease-activated PSTAGylated BiTE (PAPB), is composed of four molecules: a shielding polypeptide domain (PSTAG), a protease-activated linker (PLGLAG), and a BiTE core comprised of two different scFv sequences. The scFv segments are derived from durvalumab and blinatumomab, which are mAbs against human PD-L1 and human CD3, respectively. PD-L1 is a tumor-associated antigen targeted by the first scFv, and CD3 is a specific T cell marker targeted by another presumed scFv antibody. PSTAG reduces the interaction of BiTE with nontumor cells and thereby improves its half-life, whereas PLGLAG, when cleaved by matrix metalloproteinase-2 (MMP2), augments the BiTE core to bind PD-L1 and CD3 in the tumor microenvironment and exerts its antitumor activity. In experimental mice, the plasma half-life of PAPB was found to be greater than that of BiTE core alone and extended from 2.46 to 6.34 hr, implying the role of PSTAG. In mice subjected to melanoma (A375) xenografts, PAPB induces marked T cell infiltration in the tumor environment and impedes its proliferation without triggering T cells in the peripheral blood.

Moreover, a recently evolved practice called “hyperthermia therapy” involves the conjugation of small antibody fragments with magnetic nanoparticles (MNPs). These selectively target tumor cells and their associated antigens. Rezaei et al. [97] constructed a murine-derived scFv as a novel anti-CD47 variant that is expressed on the surface of most cancer cells. The anti-CD47 scFv conjugated with MNP was examined by magnetic activated cell sorting (MACS) for its activity against CD47+ on surface cancer cells. As a result, the anti-CD47 scFv MNP conjugate experiment indicated that the fragment antibodies were bound to CD47+ but not to cells incubated with nonconjugated scFv. The Prussian blue staining confirmed CD47 protein recognition by anti-CD47 scFv-fMA conjugate on the bladder cell lines but was negative for HepG2 control cell lines without CD47. Furthermore, the specific targeting of anti-CD47 scFv-fMA conjugate along with an external magnetic field increased the deaths of bladder cell lines but not HepG2 control cells. Hence, this practice will be an effective tool for cancer cell thermotherapy and bionano-based targeting techniques for early detection of tumors in the coming years.

Engineered small antibody fragments have also been conjugated with cytotoxic drugs to guide the selective effector function of those drugs. Fibroblast growth factor receptor 2 (FGFR2) is a molecule overexpressed in numerous tumor cells but less on healthy cell surfaces. In light of this, Borek et al. [98] created a scFv antibody against the FGFR2 receptor using the Tomlinson I and J libraries, then reformatted the conjugate into a bivalent diabody known as a scFv-Fc anti-FGFR2 antibody. In subsequent analysis, this conjugate displayed the highest affinity for FGFR2 with a KD of 0.76 nM. Furthermore, it appears to be selectively internalized into human gastric and colorectal cancer cell lines overexpressing FGFR2, such as Snu-16 and NCI-H716, respectively, under confocal microscopy. Keeping this in mind, the construct was then conjugated to the cytotoxic drug monomethyl-auristatin E (vcMMAE) via cysteine residue as “scFvF7-Fc-vcMMAE.” As a result, the selective cytotoxicity of vcMMAE against Snu-16 and NCI-H716 was enhanced greatly by half-maximal inhibitory (IC50) values of 0.89 and 7.7 nM, respectively. But the U2OS cell line negative for FGFR2 displayed a limited sensitivity, and none of them are damaged by unconjugated scFvF7-Fc, implying that scFv is carried out MMAE to FGFR2-positive tumor cells. Thus, the so-called cytotoxic drug selectively targets tumor cells with FGFR2 overexpression. Likewise, an antifibroblast growth factor 2 (FGF2) IgG mAb, named 3F12E7 mAb, has been used to generate a scFv antibody that selectively targets FGF2 in tumor extracts. The 3F12E7 scFv construct deters tumor growth at a comparable level to a full-sized IgG complement in an experimental model, though it is prone to aggregation [99].

Cell adhesion molecule 1 (CADM1) from an Ig superfamily member was also reported to have tumor suppressor activity in numerous cancer forms like ovarian [100], breast [101], and pancreatic [102], and in all those cases, the loss of CADM1 expression was recognized as a marker of tumor progression and metastasis. However, this molecule is exceptionally overexpressed in small-cell lung cancer, which is an extremely invasive neuroendocrine carcinoma that metastasizes faster and has a poor prognosis once established [103]. In those small lung cancer cells, CADM1 specifically expresses itself in the form of a membrane-bound fragment of CADM1, which is induced by proteolytic membrane proximal cleavage of CADM1 with protease enzymes such as ADAM10 and secretase. Thus, MF-CADM1 expression on small cell lung tumor cells implies its role in promoting oncogenicity [104], and it is thus identified as a therapeutic target in those cancers. Lee et al. [49] constructed a scFv from human and mouse synthetic libraries as MF-hCADM1 and MF-mCADM1, respectively. These scFvs were then conjugated to a carrier protein, BSA to become MF-hCADM1-BSA and MF-mCADM1-BSA. The scFv antibodies that exhibited a strong affinity toward MF-hCADM1-BSA and MF-mCADM1-BSA were selected and redesigned as human IgG1-scFv-Fc antibodies. After rigorous *in vitro* evaluation and surface plasma resonance kinetics analysis of Ab–Ag interaction, the scFv-Fc antibody K103.3 was selected as a principal antibody clone, since it powerfully kills human small cell cancer cell lines including NCI-H69, NCI-H146, and NCI-H187 via activation of Jurkat T cells without severe endothelial toxicity. This scheme is promising for T-cell mediated killing of cancer in the lung and related visceral organs.

Furthermore, small antibody fragments were fused with cellular molecules that have properties of angiogenesis inhibition, immunomodulatory activity, and the recruitment of tumor-specific activated T cells to the sites of the tumor microenvironment. Hence, scFv fused with cytokines is guided directly to the targeted tumor cells against tumor-specific antigens and causes a complete regression of tumor progression [105]. In the study by Ahmadzadeh et al. [51], IP-10 was fused with a scFv antibody, which targets one of the EFGRs called HER2 that is overexpressed in breast cancer. As a result, the IP-10 (anti-HER2 scFv) fusion protein discriminately reduced cell viability in human breast carcinoma cell lines overexpressing HER2 (SK-BR-3). Moreover, a chemotactic activity assay carried out by transwell migration reveals the migration of CD8+ T cells toward the HER2+ site, implying this fusion protein will be used as an adjuvant in the future together with an HER2-based vaccine for the elicitation of immunity in the tumorigenic area [52].

#### 3.1.2. Neurodegenerative Disorders

Immunotherapy approaches in human clinical trials with therapeutic antibiotics alone or in combination with acclaimed drugs have been claimed as valuable biologics. This is because of their high specificity, low toxicity, and rapid recognition of target molecules. Alzheimer's disease is a disorder stimulated by the aggregation of the amyloid peptide (A*β*) in the brain and forms a supramacromolecular species of various sized oligomers, fibrils, and plaques. A scFv derived from a mAb bapineuzumab as scFv-h3D6 was assessed for an effect against this amyloid-peptide molecule. In an experiment, the triple transgenic mouse model of Alzheimer's disease (3xTg-AD) was used to evaluate the behavioral, cellular, and molecular levels. A stat intraperitoneal administration of so-called scFv reversed the swimming speed of mice to a normal level and substantially improved their learning and memory deficits, along with a reduction in A*β* oligomers. Though, like a full mAb, the scFv-h3D6 does not trigger the microglia sensitivity, it diminishes the typical inflammation of the experimental mouse model significantly to values acknowledged as nonpathological [106]. Moreover, scFv-h3D6 therapy altered the apolipoprotein E (apoE) and apolipoprotein J (apoJ) pathological levels at the initial phases of the disease condition [107]. A recent study also proposed A*β*O-specific scFv antibody using adenovirus vector-mediated gene delivery as a potential therapy scheme for Alzheimer's disease [108]. Moreover, a viral vector-driven brain-penetrating scFv antibody has been tested for its activity against a tubulin-associated unit peptide, 3RTau (3 repeat Tau species), that often accumulates in Alzheimer's and Pick diseases. As a result, an engineered protein, anti-3RTau scFV (3RT), has substantially reduced the accumulation of 3RTau in experimental mice overexpressing 3RTau. This was manifested by enhancements in behavioral deficits and markers of neurodegeneration accompanied by neuronal loss and astrogliosis [109].

#### 3.1.3. Autoimmune Diseases and Transplant Rejections

In autoimmune diseases, no chemotherapy approaches have been found effective to absolutely alleviate the consequences of these disorders. Like neurodegenerative disorders' therapy schemes, their management strategies are to relieve and lessen disturbing symptoms [110]. Currently, immunotherapy approaches with small biologics like antibody fragments are designed to target inflammatory cytokines, intracellular kinases, and other immune cells. These revolutionized the treatment of autoimmune diseases such as psoriasis, rheumatoid arthritis, ankylosing spondylitis, etc. However, the therapeutic efficacy and safety margins of these molecules are still requiring further standardization [111].

Thymus cell antigen 1 (THY-1) is a cell surface protein that is highly conserved and expressed on the surfaces of fibroblasts. This protein appeared as an aggregate in the synovia of rheumatoid arthritis (RA) patients. Thus, it is identified as a potential target for the design of therapy for RA. Hence, anti-THY-1 scFv antibodies have been assayed in collagen-induced arthritic mice and demonstrated to suppress the expression of JUNB (the Jun B proto-oncogene) by the has-circ-0094342/miRNA-155-5P/SPI1 axis, thereby deterring the angiogenesis of RA and differentiation of osteoclasts [112]. Likewise, a monovalent scFv antibody modified through tetramerization with the p53 tetrameric domain as scFv anticyclic citrullinated peptide antibodies (TeAb-CCP) significantly inhibited PBMCs of RA. It repressed fibroblast like synoviocytes (FLS) activation, proliferation, migration, and invasion *in vitro* by the targeted influence of citrullinated modified self-epitopes [40].

Another protein called C4d, found in most blood group-incompatible (ABOi) allograft tissues, is identified as a marker for antibody-mediated rejection (ABMR). Based on these, a scFv antibody has been used to generate CAR-regulatory T cells (CAR Tregs). The developed anti-C4d CAR-Tregs with anti-C4d scFv clones have been evaluated for their therapeutic potential against C4d for both ABMR and ABOi transplantation. An adoptive transfer of anti-C4d CAR Tregs against ABMR in ABOi-triggered mice via heart transplantation (ABOi heart allograft) resulted in a noticeable extension of the survival of mice compared to the phosphate-buffered saline (PBS) control and CAR Tregs control. This implies anti-C4d CAR Treg antibodies are a promising therapy for suppressing ABMR and improving ABOi heart allograft survival in the future [79].

#### 3.1.4. Infectious Diseases

In contemporary medicine, research on passive immunotherapy-based approaches with antibody fragments conveys encouraging outcomes for substituting chemical therapeutics for infectious diseases. Engineered antibody formats like scFv would hold the complete antigen-binding capability of the original antibody. Hence, these features could possibly permit that unique molecule to be used in the recognition of pathogens, toxins, or surface antigens. In *Corynebacterium diphtheria*, neutralizing DT by polyclonal antibodies derived from immunized animals as diphtheria antitoxin (DAT) is restricted by issues such as the risk of an allergic reaction, serum sickness, and anaphylaxis. Hence, an alternative recombinant antibody derived from B cells of immunized human volunteers as diphtheria toxoid-specific anti-DT scFvs was evaluated for its neutralizing capacity against diphtheria toxin on the Vero cell assay. As a result, the designed soluble scFv antibody showed a neutralizing activity against a twofold cytotoxic dose of diphtheria toxin, with an affinity constant of almost 107 M^−1^ [113].

Another design of scFv from a human library was tested against active methicillin-resistant *S. aureus* (MRSA) cells both *in vitro* and *in vivo*. The antibacterial activity of the fragment molecule was evaluated in three various growth settings, including human PBMCs with plasma, whole blood, and biofilm. In an *in vitro* inhibition assay, the antibacterial activity of the three screened scFv antibodies with unique sequences, including MEH63, MEH158, and MEH183, displayed significant binding to *S. aureus* and lessened its viability. Furthermore, the therapeutic efficacy of the three anti-*S. aureus* scFvs tested alone and in combination with vancomycin antibiotics produces an additive effect against *S. aureus* infections. A diminished bacterial load in the blood and reduced damage and inflammation in the tissue of the MRSA-infected mouse model challenged by the screened scFv antibodies were observed and resulting in a 100% survival rate for the positive control group compared with the negative control group [114].

A scFv antibody enriched to target a multifunctional protein called alpha-enolase (Eno1) of *Candida albicans* to pursue new therapeutic schemes with the aim of countering the emergence of resistance to its drug azoles was promising. A scFv mAb (CaS1) created via phage display recognized both recombinant CaEno1 (rCaEno1) and native CaEno1, which are expressed, respectively, by C. *albicans and* C. *tropicalis*. In an *in vivo* assay of candidiasis model mice, CaS1 actively diminished the growth of *C. albicans* and disrupted CaEno1 binding to plasminogen. As a result, the therapy prolongs the survival time of the mice by reducing the fungal load and diminishing the level of inflammatory cytokines, suggesting that CaS1 could be a promising candidate for *C*. *albicans*-specific therapy in the future [115].

During infection with the severe acute respiratory syndrome coronavirus (SARS-CoV-2), a homotrimer spike protein made of two domains, S1 and S2, plays a specific interaction role between the virus and host membrane. S1 interacts with human angiotensin converting enzyme 2 (ACE2) via its receptor domain to induce viral adhesion to the host cell, whereas the S2 domain then triggers the fusion of the host and virus membranes to allow virus entry. The binding of S1 to the ACE2 receptor initiates cleavage between the S1 and S2 domains (S1/S2 cleavage site) by the host protease furin, followed by a proteolytic cleavage at the S2′ site found upstream of the S2 domain-containing fusion peptide by transmembrane protease serine 2 (TMPRSS2). Anti-S2ʹ SARS-CoV-2 scFv antibodies targeting an epitope incorporating the S2′ cleavage site, which is conserved among variants of coronaviruses as a 12-mer linear epitope (RSFIEDLLFNKV), were designed and assayed *in vitro* and on the mammalian cell surface. An overall mechanistic model proposed with structural and biochemical schemes discloses that scFv-S2′ epitope interaction and underlines that scFvs mediate the hindering of virus entry into the host cell by limiting the access of TMPRSS2 and subsequently inhibiting the competitive cleavage at the S2′ site. Thus, the study showed a potential therapeutic promise against SARS-CoV-2 and associated infections by impeding virus entry into the host cell via targeting a conserved linear epitope [116].

### 3.2. Diagnostics

#### 3.2.1. Molecular Bioimaging

Endoscopy (colonoscopy and bronchoscopy), ultrasonography, X-ray, etc., conventional imaging techniques have been used to direct therapeutic schemes and indicate the prognosis of various disorders. But the sensitivity and accuracy of these tools at molecular level are limited and some of them are tissue-invasive. Early diagnosis also remains the main defect of most imaging techniques, which are noninvasive too (e.g., ultrasound, magnetic resonance imaging, X-rays, and nuclear imaging). Thus, molecular imaging has emerged as an ideal scheme for early and noninvasive detection of various diseases with improved insights at the biochemical and molecular levels. The advanced imaging techniques include optical (bioluminescence and fluorescence), magnetic resonance imaging (MRI), and nuclear imaging (single-photon emission computed tomography and positron emission tomography) [117, 118]. They are high-throughput and produce high-resolution images, yet extracting molecular information from them is difficult and conducted only by highly skilled physicians. Hence, small-sized antibody fragments, such as scFv antibody-based probing, are investigated as an alternative choice. Screened scFv fragments targeting colon cancer secreted protein-2 (CCSP-2) were designed as conjugates with fluorescein isothiocyanate (FITC) to be anti-CCSP-2 scFv-FITC. Thus, CCSP-2, which is highly expressed in adenocarcinoma cells, was targeted by anti-CCSP-2 scFv-FITC and proven to be a feasible molecular imaging tool that differentiates malignant tissues from nonmalignant tissues in human colon cancer. Another study carried out by Liu et al. [117] in colitis model rabbits disclosed the activity of colon inflammation via a scintigraphy probe of ^99 m^Tc-labeled scFv-VCAM-1 (vascular cell adhesion molecule-1) as a molecular image and generated a potential scheme for diagnosis of inflammatory bowel disease and immunohistochemistry analysis of inflammation.

A scFv antibody design against thymocyte differentiation antigen (Thy1) on tumor cells was constructed and expressed in three different pET32b vector backbones: pET32b-1XHis-scFv, pET32b-3XHis-scFv, and pET32b-5XHis-scFv. The Trx- and S-tags and tandem His-tags fused with anti-Thy1-scFv are purification tags that improve the solubility of the underlying construct. The pure Thy1-scFv, then conjugated to an ultrasound contrast agent (MB_Thy1-scFv_), successfully displayed enhanced molecular imaging *in vivo* on a transgenic mouse model with pancreatic ductal adenocarcinoma (PDAC) compared to a nontargeted control [75]. Likely, dually designed human and murine anti-Thy1-scFv conjugated to gas-filled microbubbles as MB_Thy1-scFv_ successfully detects PDAC both in orthotropic human xenografts and transgenic mice *in vivo* [119]. Hence, this tumor-specific scFv-guided probe is promising to uncover mysterious tumor microenvironments and will be used as an effective vehicle to deliver therapeutic molecules to tumor site specimens.

In most cases, traditional imaging approaches are nontarget and less sensitive in detecting disease stages. This may possibly mislead physicians' decisions and make them unaware of carrying out further investigations for early diagnosis. But scFv antibody-based imaging is directed against a definite antigen. Therefore, they allow the rapid diagnosis of pathological states, and due to their fast clearance from the blood, they are used as an imaging probe [75]. Not only cancerous cell antigens, but scFv antibodies are also reported against some bacterial surface antigens. For instance, 6xHis-tagged scFv fragments have been demonstrated to bind model bacterial-specific surface proteins, *Propionibacterium acnes* and *Pseudomonas aeruginosa* [120]. Thus, if these antibody fragments are designed to create a sensitive detection probe via fusion with fluorescent proteins or enzymes, they will also become useful particles for the diagnosis of drug-resistant pathogens and related biofilms. Likely, as there is an infinite repertoire of cellular and synthetic libraries, scFv-based molecular bioimaging can improve the diagnostic and therapeutic applications of modern treatment and precision medicine.

#### 3.2.2. Detection of Antimicrobial Residuals

The imprudent and widespread use of antimicrobials in animal-derived foods has been reported to cause deleterious effects on mammalian health and ecology, including the development of bacterial resistance [121]. Countries, organizations, and consortiums worldwide have established maximum residue limits for these substances in food of animal origin to prevent their residual accumulation. Different physicochemical techniques, such as chromatography alone or in tandem with other assay tools, have been recognized to be valid, sensitive, reliable, and high-throughput for the detection of various antimicrobial residues. But their application in rapid screening and field work is unsuitable since they require expensive apparatus, long-term operation, and highly skilled personnel [122]. Likely, immunological methods such as indirect competitive enzyme-linked immunosorbent (IC-ELISA) and fluorescence polarization immunoassays are limited by the inability to process many samples at once [122, 123]. Optical immunosensors, surface plasmon resonance, and fluorescent biosensors-based nanoparticles are currently being developed for the detection of multiple antibiotics [124, 125, 126, 127]. In all these cases, specific antibodies are used as biorecognition elements on the surface of sensor materials to enable the detection of those antibiotics. This is either via conjugation to quantum dots (QDs) that emit different wavelengths as detection probes (QD-Ab) or other signal probe materials that enhance simultaneous detection of different antibiotic residues [128, 129]. However, all these are too expensive and are operated only by authorized personnel. Hence, simple, robust, and cost-effective technology for the detection of antimicrobial residues was required. A conjugation of scFv with IC-ELISA is one of the reliable approaches that has allowed for easy detection of antimicrobials in some of the current investigations. For instance, scFv against ciprofloxacin (CIP) was successfully constructed through phage display and directional evolution, with a library capacity of 3.34 × 10^9^ CFU/mL obtained from *E. coli* TG1 cells. After four rounds of biopanning, about 25 phage colonies were selected randomly, and the scFv-22 clone was selected for further study due to its stable and high-binding expression in *E. coli* HB2151. Recognition mechanism analysis carried out through molecular docking with these clones showed the Val160 amino acid residue as a specific binder of scFv to CIP. In virtual mutation analysis, when Ser replaced Val160 for the generation of the mutant scFv-CIP antibody, an improved intermolecular force holding the two molecules resulted. This implies that anti-CIP scFv was highly sensitive for the rapid detection of residual CIP in animal-derived edible tissues such as beef, pork, milk, and chicken samples [42]. By the same token, a scFv against the antibiotic Norfloxacin [31] and a scFv specific for Ampicillin was effectively constructed from hybridoma cells as an alkaline phosphatase fusion protein [130].

A scFv-based hybrid gene was also generated to allow simultaneous detection of three antibiotics, including chloramphenicol (CAP), ciprofloxacin (CPFX), and sulfadimidin (SM2). The hybrid of three genes as a single CCS gene therefore constitutes CAP-ScFv, CPFX-ScFv, and SM2-ScFv. The CCS genes are assembled by PCR with three pairs of primers, and the resulting PCR product is then subcloned into a recombinant plasmid (pGEX) as pGEX-CCS and expressed in *E. coli* BL21 (DE3) cells cultured in Amp+/LB medium overnight at 37°C. The glutathione-S-transferase (GST) tag was fused with the hybrid gene CCS and expressed in pGEX-6P-1 as GST-CCS. When the construct is assayed for immunologic activity and detection of the three veterinary drug residues, it has generated a promising result, implying the GST-CCS as a rapid screening biologic for the simultaneous detection of targeted antibiotics in the future [131]. Another design of scFv antibody was also immobilized on the surface of carboxylic acid magnetic beads and effectively captured maduramicin antibiotic residue from chicken muscle with high sensitivity and specificity [132].

#### 3.2.3. Food Immunoanalysis

Food sources can mistakenly and/or intentionally mix with a trace quantity of hazardous substances, such as toxins, allergens, illegal additives, pesticides, and chemicals. These pose a threat to the health of consumers. Conventional analytical protocols by chromatographic instruments are presumed to be a golden standard due to their high specificity, sensitivity, and accuracy. However, their instrumentation requires expensive settings, highly skilled personnel, and a long-term detection process [133]. The culture methods used are limited to viable bacteria only and cannot dictate their toxins. Immunoassay methods such as ELISAs, fluorescence systems, chemiluminescence, lateral flow, and immune-PCR protocols are currently accredited as prominent analytics for the early detection of various hazards in food samples. Yet, their sensitivity and accuracy are limited by the complexity and variability of matrix compounds in food samples [133, 134]. Hence, designing improved analytical tools that are fast, reliable, sensitive, and cost-effective is a present hotspot of research to ensure the safety and quality of foods. Recently, diagnostic assays have evolved into point-of-care testing (POCT) devices [135]. The devices are designed to be portable, operate with a small sample volume, run in fewer experimental steps, decrease analytical time, and be capable of being handled by semiskilled personnel. Nevertheless, most of those POCT devises, including nanopaper-based biosensors, electrochemical lateral flow platforms, microfluidic-based devices, etc., are developed mainly for the detection of pathogen biomarkers, and their full application in food immunoanalysis is insufficient [135, 136, 137, 138]. POCT devices use a signal that enables rapid detection of analytes in food as fluorescent, colorimetric, and electrochemical indicators. Yet, reproducibility, limit of detection, and multiplex functionality of these devices need an improvement. Hence, their applicability in different food samples is still scarce. Currently, research works focus on integrating signal amplification systems into paper, chip, and nanomaterial-based devices to increase sensitivity, quantification, and multiplex detection of those devices for more effective analysis of food [135]. For instance, the use of dual-working electrodes on a lateral flow immunoassay has improved 15-fold percentage relative standard deviation reproducibility of detection of the allergen *β*-lactoglobulin in milk samples compared to a single electrode format [139]. Moreover, to overcome thermal stability problems associated with antibody structure, aptamer-based biosensors are the current area of research for food immunoanalysis. Aptamers can be labeled with multiple nanomaterials and fluorescence probes to allow simultaneous detection of multiple targets with high specificity. Hence, dual-ratio electrochemical aptasensors based on carbon nanohorns/anthraquinone-2-carboxylic acid/Au nanoparticles have been demonstrated to simultaneously detect malathion and omethoate in fruit samples (with a linear range of detection from 3 pg/mL to 3 ng/mL for malathion and from 10 pg/mL to 10 ng/mL for omethoate) [140]. Likely, hairpin DNA-assisted dual-ratiometric electrochemical aptasensors were confirmed to have significant detection of multiple mycotoxins, including aflatoxin B1 and ochratoxin A, with high sensitivity, reliability, and anti-interference ability [141]. Besides, ratiometric dual-signal electrochemical aptasensors operate in hybridization chain reaction amplification along a series of aptasensors; ferrocene and methylene blue-tagged ssDNA have proven high sensitivity for on-the-spot detection of aflatoxin B1 [142]. Although aptasensors have such potentials, still their stability and specificity during POCT detection is challenging. Hence, preparation of specific recognition molecules with distinctive specificity and low molecule weight recently attracted the attention of researchers in the field of immunoassays. In a study by Arola et al. [143] to detect two mycotoxins, HT-2 toxin (HT-2) and T-2 toxin (T-2) differentially, the authors developed an HT-2 toxin-specific simple ELISA format-based anti-immune complex (IC) scFv antibody that is fused with alkaline phosphatase. For the assay, three different samples of wheat, barley, and oats were added together with the scFv-AP antibody to an ELISA plate that was coated with the primary antibody. The response was read 15 min after incubation, with limits of detection values of 0.3 ng/mL (13 *µ*g/kg), 0.1 ng/mL (4 *µ*g/kg), and 0.3 ng/mL (16 *µ*g/kg) for the three acknowledged samples, respectively. Anti-IC scFv was specifically recognized as an IC between a primary anti-HT-2 toxin Fab fragment and HT-2 toxin, but competitive ELISA with the primary antibody only recognizes both mycotoxin molecules. This suggests that anti-IC scFv makes the assay specific for HT-2 toxin only and so can be used as a screening tool for HT-2 toxin found in food sources. In another study by Rangnoi et al. [144], chain-shuffling using a naive human library generated human antiaflatoxin B1 (AFB1) scFv antibody (yAFB1-c3) by the phage-display method. In the designed VH/VL chain-shuffled library, the clone assigned as sAFH-3e3 has displayed a 7.5-fold enhancement in sensitivity over the original scFv clone as per homology modeling and molecular docking analysis, where VH is found to be more significant for AFB1 binding over VL. Moreover, scFv antibodies tagged with enzymes like alkaline phosphatase as a fusion protein (ALP-scFv) were used in direct competitive ELISA, colorimetric enzyme immunoassay (CEIA), and chemiluminescent enzyme immunoassay (CLEIA) for reliable and sensitive detection of mycotoxins like deoxynivalenol [145], and zearalenone [146] in cereals and agro-products, respectively. A specific scFv antibody called scFv-32, screened from hybridoma cell lines by phage display, has also demonstrated its ability to recognize phenylethanolamine A (PEEA) [147]. PEEA is a newly emerged *β*-adrenergic agonist that is illegally used as a feed additive to increase carcass leanness in some countries. Residual PEAA in foods of animal origin is assumed to potentially harm the cardiovascular and central nervous systems of consumers. So, it needs early detection, where PEAA-specific scFv can be a possible diagnostic molecule in the future.

#### 3.2.4. Biotoxins Detection

Biotoxins are toxins that have biological origins and are produced by every living organism, including pathogenic microorganisms (e.g., mycotoxins, endotoxins, exotoxins, neurotoxins, etc.), plants (phytotoxins), animals (zootoxins), insects (e.g., diamphotoxin), and marine organisms (e.g., domoic acid, gymnodimines, saxitoxin, cylindrospermopsin, etc.) [148, 149, 150]. Consumption of any food or drink contaminated by one or more of these biotoxins will cause various illnesses and poisoning syndromes. Various detection techniques have been employed, as these biotoxins vary in their origin, chemical structure, and means of toxicity. Compared to traditional assay methods such as ELISA, high-performance liquid chromatography (HPLC), liquid chromatography with tandem mass spectrometry (LC-MS/MS), and mouse bioassay, immunosensors and aptasensors have evolved as alternative candidates for the detection of biotoxins from various matrices [148, 149, 150, 151]. Immunosensors employed traditional antibodies and their derivatives as the capture probes and are still considered the golden standard because of the intrinsic feature of antibodies in binding their targets with high affinity and specificity. However, they are still suffering from size, stability, conjugation chemistry, and the cost of design. A single-stranded short oligonucleotide called aptamers presents some merits as capture probes over the prevailing constraints of immunosensors [152]. But both of these methods should not be designated for competing, perhaps perceived as complementary. In both cases, interaction between sensor surface and capture probe as well as transducer (which convert analyte into measurable signal) influences the sensitivity and selectivity of detection [153].

Aptasensors are high-throughput, highly specific, cost-effective, and extremely sensitive to detect biotoxins from the nM to the fM level of limit [149]. They can easily synthesize in artificial settings and modified to a state which allows their tight binding to target molecules. Aptasensors hybridized with noble metal nanomaterials, exhibit a promising result for detection of microbial toxins [152]. Nanoparticle-based sensors such as gold nanoparticles, magnetic nanoparticles, quantum dots, peptide nanotubes, etc., have also demonstrated to have a range of application in detection of pathogens and their toxins as well as veterinary drugs in foods of animal origin [154, 155]. Cell-based biosensors have also been identified as good platforms, but their specificity is low and limited to living cells that are directly integrated into biosensor materials [156]. Hence, upon exposure to presumed toxins, they read out cellular responses as signals transduced by secondary transducers. Furthermore, antibody-based detection methods, particularly for biotoxins originated from pathogenic microorganisms with small antibody fragments, have been attracted the attention of many scholars since a few years ago. Chen et al. [37] have designed a tetravalent anti-*Staphylococcal* enterotoxin A (SEA) antibody gene via a tetramerization scheme by linking the human p53 tetramerization domain with the carboxyl terminus of the anti-SEA-scFv and purifying it from *E*. *coli* BL21. This tetravalent antibody has been shown to display specific binding activity and sensitivity toward SEA in an indirect, noncompetitive ELISA as compared to the scFv monomer in the sandwich ELISA assay. Molecular docking analysis of this fragment molecule showed, its specific interaction with SEA is via the opposite side of the residue linked to p53. Mechaly et al. [157] also constructed a chimeric scFv-Fc antibody that selectively displays an ultra-high affinity toward the 25 kDa synaptosomal associated protein (SNAP-25) neoepitope after an *in vivo* experiment conducted on mice and rabbits to detect and quantify Botulinum neurotoxin type E. Botulinum neurotoxin type E serotype (BoNT/E) is the fastest at cleaving SNAP-25 in motor neurons, causing progressive flaccid paralysis. In the human neuroblastoma cell line SiMa treated with BoNT/E, this chimeric antibody specifically recognizes the cleaved SNAP-25_1–180_ but not the intact SNAP-25_1–206_. This means the designed chimeric antibody can be used in in *vitro* assays to define the effectiveness of antitoxin preparations and thereby decrease the use of laboratory animals.

## 4. Challenges and Limitations of scFv Antibody

Although plenty of merits exist with the scFv antibody, most of the optimistic features of those biologics were found challenging. Absence of the Fc domain in scFv antibody makes the molecule less thermostable than parental mAb, and the lack of FcRn-mediated recycling prones the molecules to a short circulation half-life. Likewise, the miniature size of the biologic promotes the tendency for aggregation that can generate a molecule with an increased risk of immunogenicity. Its rapid clearance from the circulation leads to the need for higher and repeated dosing [99]. Though methods that involve the fusion of scFv with bovine serum albumin (BSA) or polyethylene glycol (PEG) are designed to improve its half-life retention, they can also outweigh the benefits of scFv over a mAb due to the increase in size of the molecule and the cost of the experiment as well. The challenges associated with the generation of full recombinant antibodies can equally apply to small fragment antibody molecules, including but not limited to technologies used to bioengineer recombinant antibodies, expression, purification, safety, and stability control platforms.

The hybridoma technology invented in 1975 for the production of mAbs has been applied with slight modifications till now. However, the utilization of only murine-derived antibodies, which elicit immunity in humans against antimurine antibodies, limited the therapeutic efficacy of recombinant molecules derived via this technology [50]. The generation of heterogonous products from the method when used to produce scFv is also erroneous, as it delivers more than one heavy and light chain from one cell line [10]. Phage display technology, which was used to generate fully human or humanized scFv antibody molecules, still suffers from experimental complexity, prolonged time, and a high production cost. The use of transgenic animals with human immunoglobulin loci integrated also requires tedious procedures to integrate recombined genes into newly created host animals [50]. Furthermore, single B-cell technology developed to generate a native human antibody with their natural combination of VL and VH domains involves screening techniques such as fluorescence-activated cell sorting (FACS) and microengraving to figure out single cells that express antibodies of interest, which are still highly expensive and tiresome processes [50, 158]. The composition of the linker peptide can also influence the physicochemical properties of the target biologics and their *in vivo* activity. Repetitive linkers are determined to cause problems associated with PCR-based approaches and immunogenicity as well. Though alternative nonrepetitive linkers have been employed for improved *in vivo* activities, they are less flexible, unlike repetitive linkers [23].

Expression systems used also restrict the conformational folding and yielding capacity of active scFv antibodies. The prokaryotic model suffers from the absence of protein glycosylation and the formation of endotoxins during the expression process that are difficult to remove from the medium, while the eukaryotic single-cell model suffers from the misfolding of molecules within the endoplasmic reticulum (ER). Though mammalian cell lines are used to mitigate limitations with protein folding and glycosylation patterns, the genetic variability between those cell lines may add different terminal glycan epitopes to recombinant proteins that are different from the natural conformation of humans, making this approach not a good platform for the production of antibody fragments [15, 16, 159]. Transgenic plant expression systems developed recently also suffer from extraction, purification procedures, and limited glycosylation patterns like microbial expression systems [160]. A baculovirus expression vector system developed also limited by posttranslational modifications with N-linked glycosylation and difficulty of transfection into insect cells as the virus infects the insect cells in a lytic manner and impedes the continuous production of the recombinant proteins in batch cultures [50, 161]. On other hand, the purification of the scFv antibody protein is more intricate because absence of Fc domain in its structure. Thus, effective purification method by Protein A or Protein G affinity chromatography is more difficult and needs a multimodal operation of diverse chromatographic and nonchromatographic interactions. Affinity tags are fused with scFv antibody for the selective capture and efficient purification, but proteolytic cleavage at the end may not completely remove the full tags and leave some residual amino acids that lead to aggregation and/or misfolding of scFv antibodies and even enhance their immunogenicity [21, 72].

## 5. Future Perspectives

Over the last decades, an excessive amount of progress has been made in the generation of novel targeting antibody molecules. The technologies used to synthesize these molecules have been evolving endlessly and will continue to do so to amend the constraints associated with their purification, safety, stability, circulation half-life, efficacy, immunogenicity, and adverse effects by means of their clinical application. With their limitations, scFv antibodies have been used for several purposes. These include the treatment or diagnosis of tumors, autoimmune diseases, and infectious diseases; the specific delivery of radioactive or chemotherapeutic payloads to different cancerous and noncancerous cells through conjugation to drugs or nanoparticles; the detection of antimicrobial residues; and the immunoassay of toxins in food. When the molecular mechanisms of a given disease are fully understood and definite cellular molecules involved during the course of their pathogenesis are markedly identified in the same way as fragment antibodies, scFv may offer effective therapeutic and diagnostic options in the near future.

Genetic fusion of the scFv antibodies with other immune molecules like cytokines or chemokines would competently direct the antibody molecules to the regions of the disease only or to the specific molecules expressed in the pathological conditions. These lessen the adverse activity of fragment molecules on healthy cells, thereby eliciting their therapeutic index by localizing their biological activity. An immunocytokine trivalent antibody fragment has been reported by fusion of TNF-*α* monomer with a scFv, where the homotrimeric assembly was simplified by the cytokine moiety of the construct. Yet no scFv antibody-based therapeutics have evolved into clinical applications for neurodegenerative diseases. Recent preclinical research is promising to raise the scFv antibodies that possibly target misfolded proteins aggregated in brains and cause Alzheimer's, Huntington's, or Parkinson's disease. Bispecific scFv-based formats are expected to overcome the blood–brain barrier (BBB) challenge by utilizing the biological transportation system of receptor-mediated transcytosis, a route that delivers essential macromolecules to the brain [109]. Thus, one binding site recognizes a receptor that assists protein molecules in crossing the BBB and getting access into the brain parenchyma, while the other binding site recognizes a target molecule to neutralize it. The scFv antibody fragments are more flexible and can be potentiated to solve efficacy problems associated with full-sized mAbs in the treatment and diagnosis of solid tumors due to their intrinsic miniaturization. Antibody molecules in general are restricted to the extracellular milieu since they cannot cross the lipid bilayer. But scFv antibodies can be possibly conjugated with nanocarriers, cell-penetrating peptides, or constructed with a sequence that enables them to recognize a receptor on the cell surface and so internalize into the target cell through receptor-mediated endocytosis. Thus, it will recognize only the target submolecules of cancer cells and relieve the arduous side effects incurred by chemotherapy and radiotherapy approaches. Therapeutic antibodies have been used as a naked antibody that targets the underlying disease microenvironments or molecules via different routes of attack, including antibody-dependent cell-mediated cytotoxicity (ADCC) or complement-dependent cytotoxicity (CDC), by stimulating the direct apoptosis of sickly cells and targeting immune checkpoints. As an engineered design, the scFv antibody was conjugated with cytokines (immunocytokines), drugs, radionuclides, liposomes (immunoliposomes), and chimeric antigen receptor T cells (scFv-CAR-T). Studies also demonstrated that scFv is amended to have a neutralizing capacity for venoms from poisonous snakebites like cobra venom [162, 163, 164]. As further neutralization assays improve from time to time, polyspecific antibody fragments, or antivenom polyclonal scFvs designs will become a potential neutralizing molecule for a complex mixture of venoms from various animal and insect bites [165].

Due to the continuous growth of recombinant antibody technology, the features of scFv antibodies, such as molecular weight, binding affinity, and valency, can be greatly amended and optimized based on the aim of application. The cost of production, postmarketing issues related to therapeutic expenses, and pharmacokinetic properties are all expected to be greatly improved, which would solve the massive economic burden on both pharmaceutical companies and patients at risk. Besides display technologies used for affinity selections, advancements in bioinformatics will enable easier analysis of induced mutations in the scFv antibody structure under study, particularly CDR, via random or hotspot mutagenesis to predict their high-affinity characteristic over the native antibody. Moreover, progress in new technological tools is expected to sharpen and enhance the therapeutic and diagnostic application of scFv antibodies. A structure-based analysis of antibody fragments using computational approaches [166] is likely improved to decipher immunoglobulin structure, and modeling of antibody variable regions may possibly enhance its affinity and stability along with its effective *de novo* engineering. By self-assembling scaffolds via the chaperna method, the multimerization of scFvs as nanoparticles [167] also engraves a promising approach for further avidity and thermostability potentiation of antibody fragments in the future. An anti-HER3 trivalent scFv, constructed via the Spy Catcher ligase system, improved affinity by 12-fold, unlike the monomeric anti-HER3 complement [168]. Another evolving approach involves the cyclization of scFv with an *enzyme sortase-A* to ligate the VH and VL pairs together in a closed state, which evidently suppresses the aggregation tendency of scFv antibodies without troubling their affinity [169].

## 6. Conclusion

This review has covered an overview of scFv antibodies and some of their formats, derived either from immune or synthetic libraries. The main advantages of scFv antibodies over full-length mAbs, with emphasis on the size and flexibility of the linker peptide, have been highlighted as well. Moreover, limitations of each expression system and challenges associated with each bioengineering technology, display system, and purification protocol have been discussed thoroughly. With continuous improvements and the emerging of comprehensive tools that disclose complex biological systems at the molecular level, scFv antibodies may become excellent biologics in the curing and diagnosis of many debilitating diseases in the near future. More rigorous studies and investments should be made to improve the efficacy, stability, and safety of these biologics, along with stringent quality control systems and optimization protocols.

## Figures and Tables

**Table 1:** scFv-antibody designs approved by FDA for the therapy of various cancer ailments.

Brand name (antibody name)	Format; target molecule	Approved	Company's name and approval year	References
Kimmtrak (Tebentafusp)	Bispecific; gp100 and CD3	Metastatic uveal melanoma	Immunocore and January 25, 2022	[80]
Vabysmo (Faricimab)	Bispecific; VEGF-A and Ang-2	DME and neovascular AMD	Genentech and January 28, 2022	[81]
Tecvayli (Teclistamab)	Bispecific; BCMA and CD3	Multiple myeloma	Johnson & Johnson and October 25, 2022	[82]
Blincyto® (Blinatumomab)	Bispecific®; CD19 and CD3	Philadelphia chromosome-negative relapsed or refractory B-cell precursor acute lymphoblastic leukemia	Amgen Inc. and December, 2014	[83, 84]
Carvykti (ciltacabtagene autoleucel/cilta-cel)	CAR T-cell; BCMA	Multiple myeloma	Johnson & Johnson and March 30, 2022	[85]
Yescarta (Axicabtagene ciloleucel)	CAR T cell; CD19-expressing cancer cells	Large B-cell lymphoma	Kite, a Gilead Company (Nasdaq: GILD) and April 1, 2022	[86]
Kymriah (Tisagenlecleucel)	CAR T cell; CD- 19 expressing B cells	Relapsed or refractory follicular lymphoma (FL)	Novartis Pharmaceuticals Corporation and May 27, 2022	[87]
Breyanzi (Lisocabtagene maraleucel)	CAR T cell; CD19-expressing cancer cells	Large B-cell lymphoma	Bristol Myers Squibb and June 24, 2022	[88]
Abecma (Idecabtagene vicleucel)	CAR T cell; BCMA	Multiple myeloma	Bristol Myers Squibb and March 26, 2021	[89]
Tecartus (Brexucabtagene autoleucel)	CAR T cell; CD19 and or BCMA	Relapsed or refractory B-cell precursor ALL	Kite Pharmaceuticals, Inc. and October 1, 2021	[90]
Beovu (Brolucizumab)	Humanized scFv; VEGF-A	DME	Novartis and October 8, 2019	[91, 92]

*Abbreviations*. AMD, age-related macular degeneration; ALL, acute lymphoblastic leukemia; Ang-2, Angiopoietin- 2; BCMA, B-cell maturation antigen; DME, diabetic macular edema; gp100, Glycoprotein 100; VEGF-A, vascular endothelial growth factor A.

## Data Availability

The processed data are available from the corresponding author upon request.
